# An Atypical Presentation of Guillain-Barré Syndrome: Bilateral Facial Nerve Palsy With Preserved Reflexes

**DOI:** 10.7759/cureus.102572

**Published:** 2026-01-29

**Authors:** Ali Al-Timimy, Zahwa Farooqui, Alia Choudhry, Anil Kapoor

**Affiliations:** 1 Internal Medicine, Kaiser Permanente San Francisco Medical Center, San Francisco, USA; 2 Internal Medicine, Flushing Hospital Medical Center, New York, USA; 3 Neurology, Flushing Hospital Medical Center, New York, USA

**Keywords:** acute inflammatory demyelinating polyradiculopathy, bilateral facial nerve enhancement, bilateral facial nerve palsy, guillain-barré syndrome, intravenous immunoglobulin (ivig)

## Abstract

Guillain-Barré syndrome (GBS) classically presents with ascending weakness and areflexia, but atypical variants can pose diagnostic challenges. Bilateral facial diplegia with paresthesia represents a rare localized variant, and the presence of preserved reflexes further deviates from typical presentations. This case highlights an unusual presentation of GBS characterized by bilateral facial nerve palsy with preserved deep tendon reflexes. We present here the case of a 29-year-old woman who presented with bilateral facial numbness and distal limb paresthesia one week after symptom onset and three weeks following Tdap (tetanus, diphtheria, acellular pertussis) vaccination. This case emphasizes that clinicians should maintain a high index of suspicion for GBS in patients presenting with bilateral facial palsy and paresthesia, even when classic areflexia is absent, as early recognition and immunotherapy can significantly improve outcomes.

## Introduction

Guillain-Barré syndrome (GBS) is an acute immune-mediated polyradiculoneuropathy affecting approximately 100,000 individuals worldwide annually. While the classic presentation features progressive ascending limb weakness with hyporeflexia or areflexia [1], GBS encompasses a clinically heterogeneous spectrum that includes localized variants such as pharyngeal-cervical-brachial weakness, bifacial weakness with paresthesias (BFP), and Miller Fisher syndrome [1,2].

BFP represents a rare localized variant characterized by rapidly progressive bilateral facial weakness without limb weakness, ataxia, or other cranial neuropathies, often accompanied by distal limb paresthesias and diminished deep tendon reflexes. This variant is frequently misdiagnosed as bilateral Bell palsy, Lyme disease, or sarcoidosis due to its rarity and atypical presentation [2]. Adding to diagnostic complexity, certain GBS subtypes may present with preserved or even exaggerated reflexes, particularly in acute motor axonal neuropathy (AMAN). Approximately 10% of AMAN patients maintain normal or hyperactive tendon reflexes throughout the disease course, contradicting the traditional diagnostic criterion of areflexia and potentially leading to delayed recognition if clinicians are unaware of this variant [3]. Diagnosis relies on clinical criteria established by the National Institute of Neurological Disorders and Stroke (NINDS) and Brighton Collaboration, requiring bilateral flaccid weakness and decreased reflexes in the absence of alternative causes. However, these criteria may not capture all variants, particularly those with localized weakness or preserved reflexes [1]. Supportive findings include cerebrospinal fluid albuminocytologic dissociation and electrodiagnostic evidence of neuropathy, though both may be normal in early stages [1].

Early recognition and treatment are critical, as approximately 30% of patients progress to respiratory failure requiring mechanical ventilation, and up to 5% die despite treatment [4]. Intravenous immunoglobulin (IVIG) and plasma exchange remain the only established immunotherapies that accelerate recovery, underscoring the importance of maintaining high diagnostic suspicion for atypical presentations [1].

## Case presentation

A 29-year-old woman presented to the emergency department with bilateral facial numbness and numbness of her hands and feet. Her symptoms started one week prior to presentation, beginning with a posterior headache, followed by numbness over her tongue, progressing to involve her entire face, hands, and feet. She mentioned that she received the Tdap (tetanus, diphtheria, acellular pertussis) vaccine about three weeks prior to her presentation.

Comprehensive metabolic panel, complete blood count and inflammatory markers were done and were unremarkable (Tables 1, 2). Urine toxicology screen was negative. On physical exam, the patient was unable to close her eyes, indicating bilateral facial nerve palsy and, interestingly, throughout the hospital course, she had preserved reflexes, with the exception of absent ankle reflex.

**Table 1:** Comprehensive metabolic panel and chemistry (blood)

Chemistry (blood)	Value	Reference range
Glucose	88	70-99 mg/dL
BUN	14	7-20 mg/dL
Creatinine	0.6	0.6-1.3 mg/dL
Sodium	137	135-145 mEq/L
Potassium	3.8	3.5-5.0 mEq/L
Chloride	105	98-106 mEq/L
Carbon dioxide	23	22-29 mEq/L
Calcium	9.5	8.6-10.2 mg/dL
Anion gap	9	8-12 mEq/L
Phosphorus	5	2.5-4.5 mg/dL
Protein, total	8	6-8.3 mg/dL
Albumin	5	3.5-5.0 mg/dL
Alanine aminotransferase (ALT)	37	7-56 U/L
Aspartate aminotransferase (AST)	34	10-40 U/L
Alkaline phosphatase	57	44-147 U/L
Magnesium	2.1	1.3-2.1 mEq/L
C-reactive protein	<0.5	<1.0 mg/dL
Procalcitonin	0.04	<0.05 ng/mL

**Table 2:** Complete blood count

Complete blood count	Value	Reference range (adult)
White blood cells	8.9	4.0–11.0 ×10³/µL
Red blood cells	4.29	4.2–5.4 ×10⁶/µL (female)
Hemoglobin	11.3	12.0–16.0 g/dL (female)
Hematocrit	33.9	36–46 % (female)
Mean corpuscular volume (MCV)	79.0	80–100 fL
Red cell distribution width (RDW)	16.5	11.5–14.5 %
Platelet count	333	150–400 ×10³/µL

Lumbar puncture was performed during her hospital stay, which showed increased protein (109 mg/dL) (Table 3). The rest of her studies were negative.

**Table 3:** Cerebrospinal fluid (CSF) analysis

Cerebrospinal fluid analysis	Value	Reference range
Appearance	Clear	Clear
Color	Colorless	Colorless
White blood cells	3	0-5 cells/µL
Red blood cells	2	0 cells/µL
Glucose	56	45-80 mg/dL
Protein	109	15-45 mg/dL

**Figure 1:**
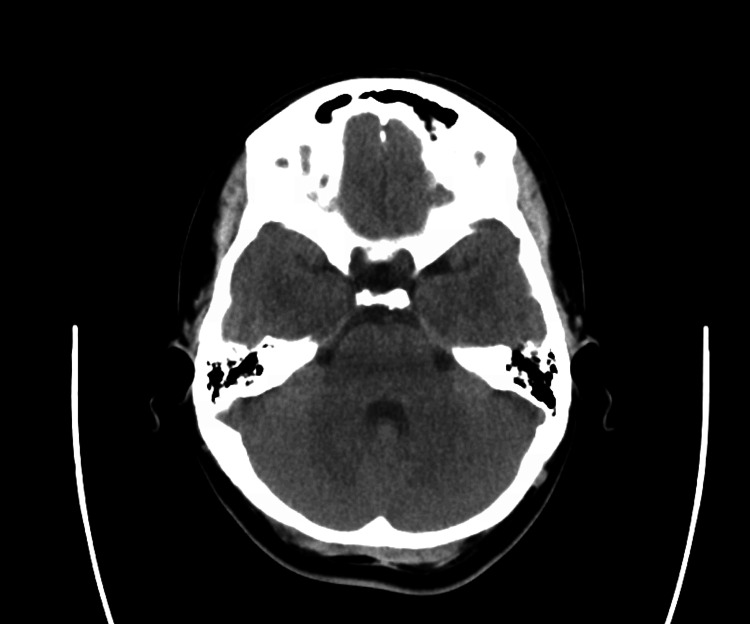
CT head (transverse view)

**Figure 2:**
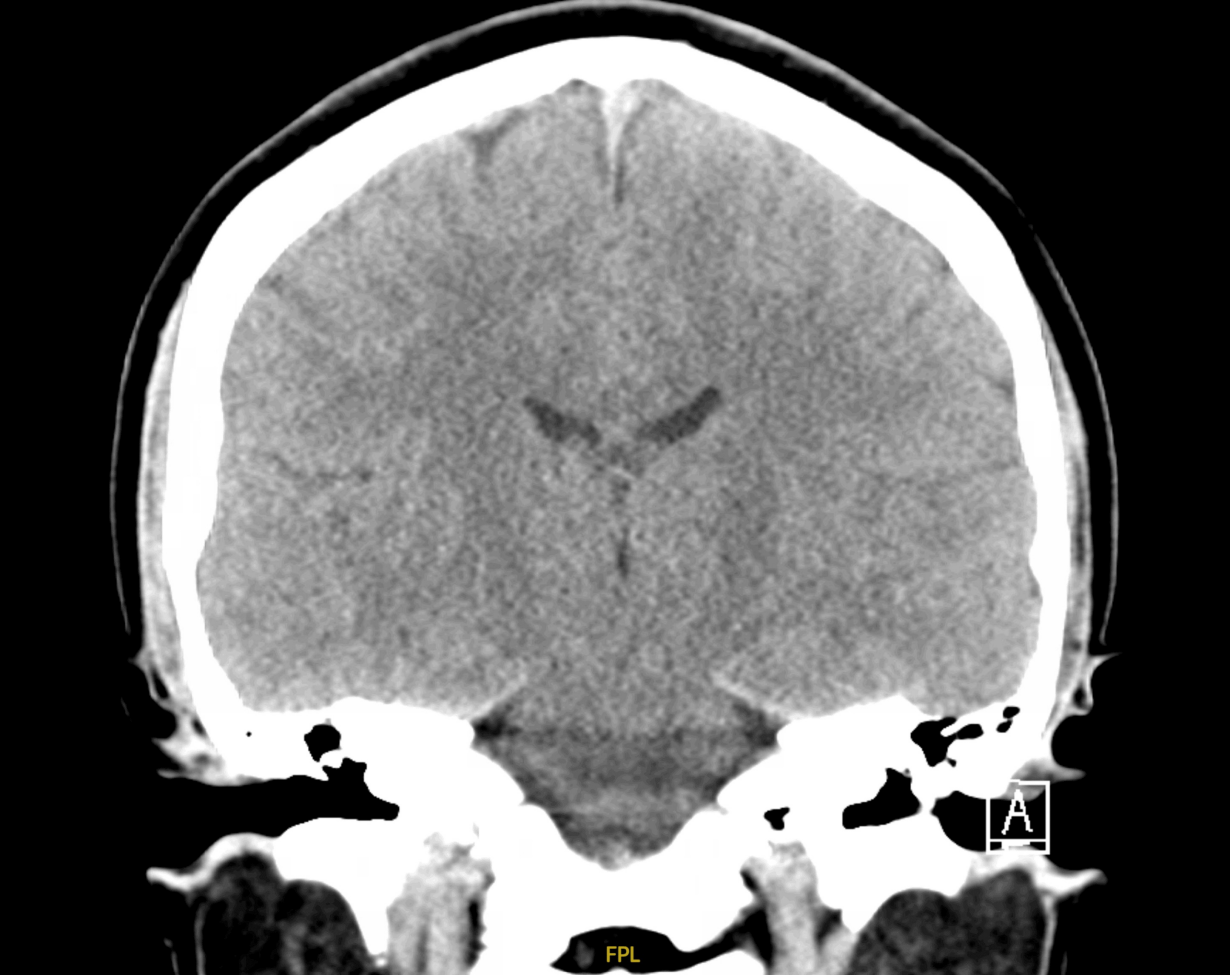
CT head (coronal view)

**Figure 3:**
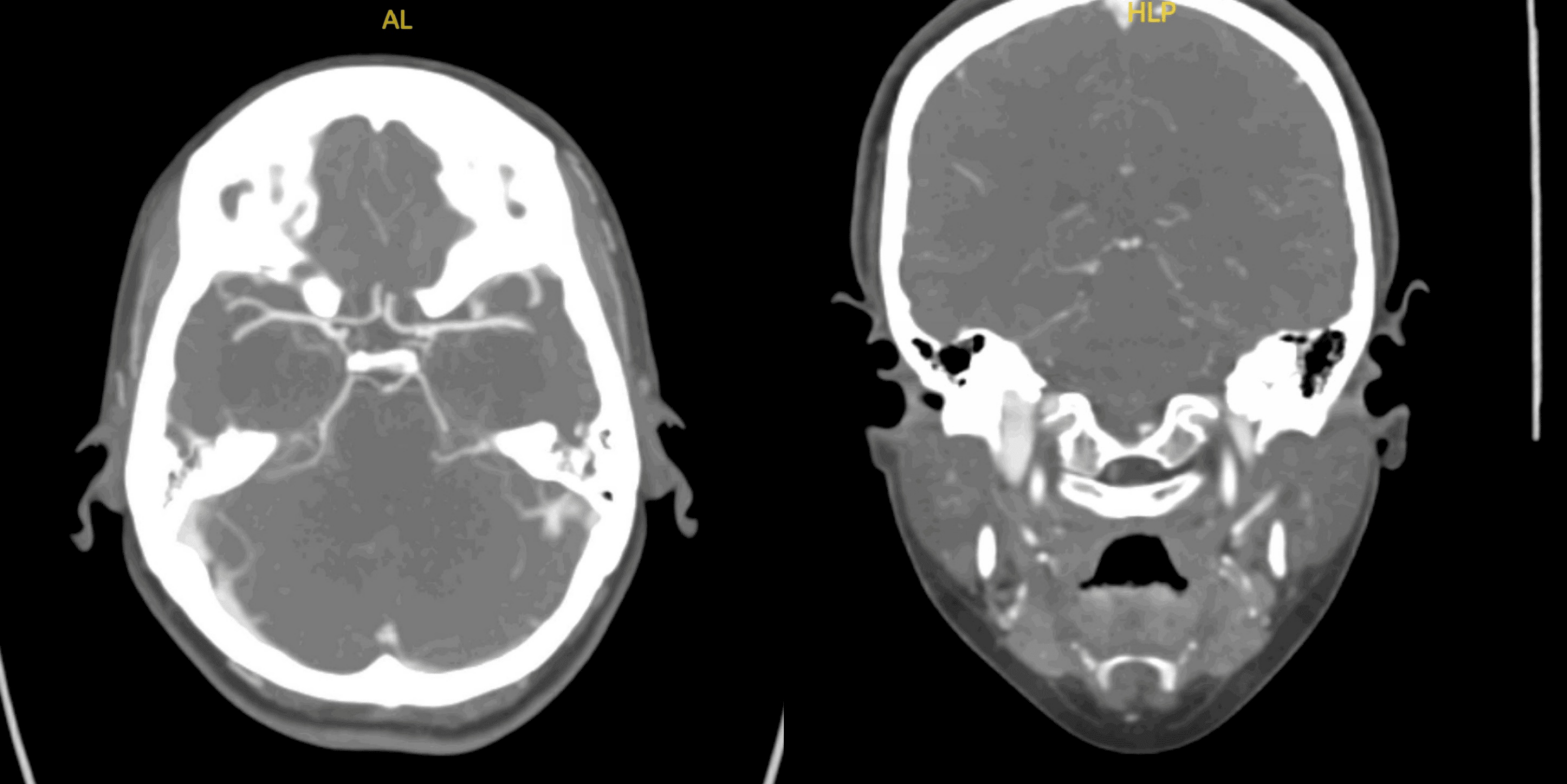
Essentially normal CT angiogram of the head and neck negative for high-grade stenosis, large vessel occlusion or aneurysm

**Figure 4:**
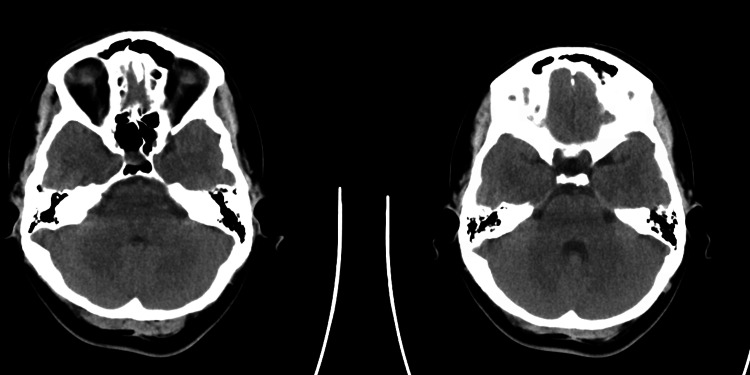
Essentially normal CT perfusion scan of the brain showing normal cerebral blood flow

**Figure 5:**
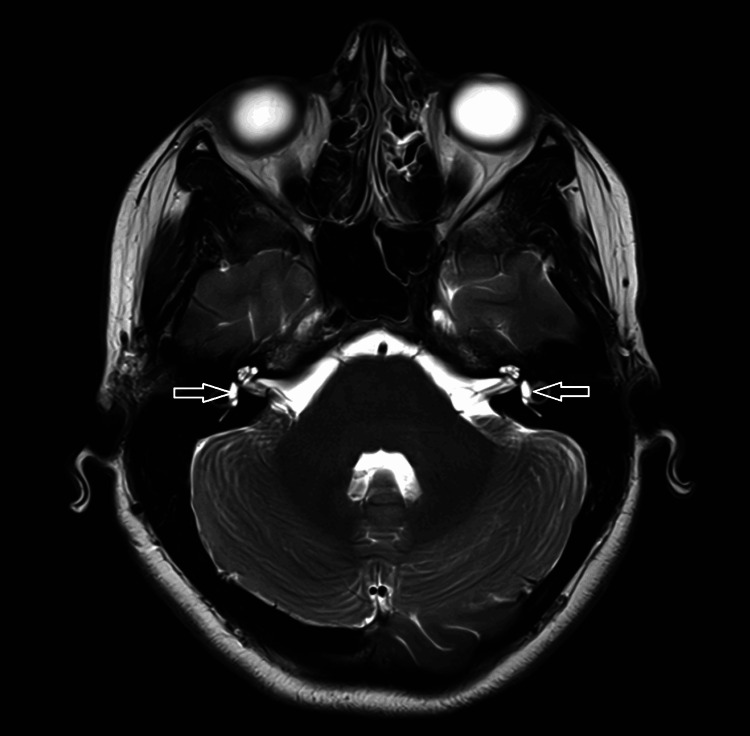
MRI brain showing bilateral facial nerve enhancement (arrows)

CT of the head, CTA head and neck, and a CT perfusion study were all negative (Figures 1-4). MRI brain was done (Figure 5), which showed bilateral enhancement of the distal meatal, labyrinthine, tympanic and mastoid segments of the facial nerves.

The patient completed 14 days of ceftriaxone and doxycycline until her Rickettsial panel resulted negative and therefore a rickettsial infection was ruled out. Electromyography (EMG) was done with nerve conduction studies carried out in various nerves in the upper and lower extremities bilaterally. The studies revealed that distal motor latencies are prolonged in bilateral median, bilateral peroneal, and bilateral tibial nerves. Amplitudes were low in most nerves examined. Conduction blocks were seen in bilateral ulnar nerves at the elbow and bilateral peroneal nerves at tibial heads. Conduction velocities were slow in both tibial nerves. F waves were prolonged in various nerves. Sensory action potentials were essentially normal with normal distal latencies except for the right median sensory action potential being absent. In summary, the study was suggestive of early acute inflammatory demyelinating polyradiculopathy (also known as Guillain-Barré syndrome), for which the patient was started on IVIG 0.4 mg/kg x 5 days.

The patient was later seen as an outpatient several weeks following her discharge with complete resolution of her symptoms and no limitation on her daily activities, having responded well to treatment. 

## Discussion

This case illustrates an atypical presentation of GBS characterized by bilateral facial nerve palsy with preserved deep tendon reflexes, highlighting the diagnostic challenges posed by variants that deviate from classic clinical criteria. While classic GBS typically presents with ascending weakness and areflexia [1], the syndrome encompasses a clinically heterogeneous spectrum that includes localized variants such as BFP, which is characterized by rapidly progressive bilateral facial weakness without limb weakness, ataxia, or other cranial neuropathies, often accompanied by distal limb paresthesias. It also discusses the possible role vaccination can play as a trigger for GBS [5]. The patient's initial diagnostic workup appropriately excluded central causes such as stroke, tumor, and demyelinating disorders. The absence of findings on brain CT and MRI, except for bilateral facial nerve enhancement, shifted focus toward peripheral nervous system involvement. Lumbar puncture findings of elevated cerebrospinal fluid protein without pleocytosis (albuminocytologic dissociation) are highly suggestive of GBS [6] and further support this diagnosis. Albuminocytologic dissociation is present in approximately 70% of GBS patients, though this proportion increases with time from weakness onset (57% at ≤4 days versus 84% after four days), and normal protein levels do not exclude the diagnosis [1]. High CSF protein levels are associated with a demyelinating subtype and early, severe disease course. Regarding cranial nerve involvement, the presence of anti-ganglioside antibodies, particularly anti-GT1a antibodies, has been implicated in the pathogenesis of cranial nerve variants of GBS [1]. Anti-GT1a antibodies, which often cross-react with GQ1b, are detected in approximately 50% of patients with pharyngeal-cervical-brachial weakness and bulbar palsy, as GT1a gangliosides are abundant on the glossopharyngeal and vagus nerves [7,8]. Bilateral facial diplegia as an initial manifestation of GBS, though rare, has been documented in the medical literature. A particularly notable feature of this case is the preservation of deep tendon reflexes throughout most of the hospital course. While areflexia is considered a traditional diagnostic criterion for GBS, certain subtypes may present with preserved or even exaggerated reflexes, particularly in AMAN. Approximately 10% of AMAN patients maintain normal or hyperactive tendon reflexes throughout the disease course, and about 5% demonstrate preserved reflexes initially that diminish only at disease peak. This preservation of reflexes contradicts traditional diagnostic criteria and can lead to delayed recognition if clinicians are unaware of this variant presentation. The patient's recent Tdap vaccination could represent a potential trigger for GBS development. While active surveillance data covering two million doses of Tdap administered to adolescent and adult populations failed to demonstrate an association between tetanus toxoid-containing vaccines and GBS within six weeks following vaccination, the temporal relationship warrants consideration [9,10]. The absolute risk of vaccine-associated GBS remains extremely low (less than 1 per million vaccinations for most vaccines), and importantly, the attributable risk of GBS with influenza infection is considerably higher (17 per million infections) than with influenza vaccination, emphasizing that vaccination benefits far outweigh this minimal risk [1,5]. EMG demonstrated early acute inflammatory demyelinating polyradiculopathy, consistent with GBS. Treatment with IVIG was initiated promptly after diagnosis confirmation, consistent with established guidelines. Both IVIG and plasma exchange are equally effective in improving disease outcome by accelerating recovery when started within 2-4 weeks of disease onset, though neither treatment halts disease progression nor alters the extent of nerve damage. IVIG at a dose of 2 g/kg administered over five days has shown efficacy in accelerating recovery and is often preferred over plasma exchange due to greater convenience and availability. In patients with autonomic dysfunction and in children, IVIG is the preferred treatment modality. Early intervention is critical, as approximately 30% of patients with GBS progress to respiratory failure requiring mechanical ventilation, and up to 5% die despite treatment [1,5,7,11].

This case also underscores the importance of considering a broad differential diagnosis for bilateral facial palsy, including Lyme disease, sarcoidosis, and other infectious etiologies. In Lyme-endemic areas, Lyme disease can cause up to 25% of facial paralysis cases, and bilateral facial nerve palsy is emerging as a common manifestation of Lyme neuroborreliosis in pediatric populations. Other important diagnostic considerations include Ramsay-Hunt syndrome, viral/bacterial CNS infections, neoplasias, autoimmune diseases, and otogenic processes. Empiric antibiotics were appropriately initiated while awaiting serologic results, demonstrating a prudent approach to ruling out serious conditions that require specific treatment [12-16]. CSF analysis has proven to be a reliable diagnostic tool for identifying Lyme neuroborreliosis (100% sensitivity) and other viral/bacterial CNS infections, while MRI with contrast is most useful for diagnosing neoplasias and otogenic processes [14].

## Conclusions

This case report highlights an atypical presentation of GBS with bilateral facial nerve palsy with preserved reflexes as the predominant feature. Early recognition and diagnosis of GBS are essential for initiating timely treatment, which can significantly influence patient outcomes. This case also illustrates the importance of a comprehensive diagnostic evaluation in patients presenting with unusual neurological symptoms. Awareness of atypical manifestations of GBS can aid clinicians in making accurate diagnoses and providing optimal care.
